# Evaluation of Coating Thickness Using Lift-Off Insensitivity of Eddy Current Sensor

**DOI:** 10.3390/s21020419

**Published:** 2021-01-09

**Authors:** Xiaobai Meng, Mingyang Lu, Wuliang Yin, Abdeldjalil Bennecer, Katherine J. Kirk

**Affiliations:** 1 Faculty of Art, Science and Technology, University of Northampton, Northampton NN1 5PH, UK; abdeldjalil.bennecer@northampton.ac.uk (A.B.); katherine.kirk@northampton.ac.uk (K.J.K.); 2 School of Electrical and Electronic Engineering, University of Manchester, Sackville Street Building, Manchester M13 9PL, UK; wuliang.yin@manchester.ac.uk

**Keywords:** multifrequency eddy current, lift-off inversion, coating thickness, nondestructive testing, multilayer conductor

## Abstract

Defect detection in ferromagnetic substrates is often hampered by nonmagnetic coating thickness variation when using conventional eddy current testing technique. The lift-off distance between the sample and the sensor is one of the main obstacles for the thickness measurement of nonmagnetic coatings on ferromagnetic substrates when using the eddy current testing technique. Based on the eddy current thin-skin effect and the lift-off insensitive inductance (LII), a simplified iterative algorithm is proposed for reducing the lift-off variation effect using a multifrequency sensor. Compared to the previous techniques on compensating the lift-off error (e.g., the lift-off point of intersection) while retrieving the thickness, the simplified inductance algorithms avoid the computation burden of integration, which are used as embedded algorithms for the online retrieval of lift-offs via each frequency channel. The LII is determined by the dimension and geometry of the sensor, thus eliminating the need for empirical calibration. The method is validated by means of experimental measurements of the inductance of coatings with different materials and thicknesses on ferrous substrates (dual-phase alloy). The error of the calculated coating thickness has been controlled to within 3% for an extended lift-off range of up to 10 mm.

## 1. Introduction

Coatings serve as protective barriers for substrate materials in industrial applications. In order to investigate their characteristics, various nondestructive techniques, chiefly eddy current (EC) sensing, have been used to directly measure the thickness of coating on a conductive substrate in a noncontact manner [1,2,3,4].

Diverse methods using EC sensors have been proposed for the measurement of coating thickness. Kim et al. reported a noncontact and on-line method using a dual EC sensor setup to reduce the measurement error of film coatings [5]. An EC testing-based method has been applied to measure the impedance of the conductive substrate and determine the coating thickness [6]. Considering the ferrous substrate, Yang and Tai have used the swept-frequency eddy-current (SFEC) for the determination of the substrate permeability, which serve as the input for subsequent measurements of conductivity and thickness of coatings using the pulsed eddy current (PEC) method [7,8,9,10,11]. Other methods include the dual-frequency EC sensing technique [12], swept-frequency [13,14,15] and single-frequency [16] eddy current sensing for the thickness measurement of nonmetallic coatings, error compensations on the thickness of conductive coatings [17], reconstruction of multilayer electromagnetic parameters [18,19], numerical models [20], and alternative strategies on monitoring the coatings [21]. The proposed techniques can cope with small lift-off variations of up to 6 mm for either magnetic or nonmagnetic materials.

In practical measurements, the sensitivity of the EC signal is frequency dependent and varies with different values of material and geometric properties (e.g., thickness) [22], which then affects the reliability and accuracy of the defect evaluation. Therefore, it is necessary to analyse the characteristics of sensor-sample using signals obtained from different frequencies using multifrequency eddy current (MEC) testing. Compared to the PEC, the MEC has better signal-to-noise ratio (SNR) particularly under high working frequencies [23]). By using multiple frequency channels [24] and curve-matching functions (e.g., polynomials), online real-time monitoring of parameters can be achieved. However, like other EC techniques, MEC can be significantly affected by coating variations that manifest in the lift-off distance between the sensor and test piece. Previously, to address the lift-off issue, a time-domain feature, the lift-off point of intersection has been used for the measurement of coating thickness based on the PEC [25].

For the MEC, previous works have been proposed to reduce the error (caused by the lift-off distance variation) to derive important parameters such as the thickness (single layer), magnetic permeability, and electrical conductivity of samples [25,26,27,28,29,30,31,32,33,34,35,36,37,38,39]. The methods involve novel sensor structure (e.g., triple-coil eddy current sensor system), compensation algorithms, and frequency features (e.g., revised/compensated peak frequency for nonmagnetic or zero-crossing frequency for ferromagnetic materials) [40,41,42,43,44,45,46]. However, a few methods have directly derived the lift-off distance. Besides, previous scenarios on reducing the error of lift-offs merely apply for a smaller range of lift-offs (mostly up to 6 mm). Moreover, previous methods merely apply to the single-layer conductive structures. For the dual-layer plates, properties of substrates (including the thickness, electrical conductivity, and magnetic permeability) significantly affect the measured signals (voltage, impedance, or inductance). Thus, alternative features are required to retrieve the thickness of coatings on ferromagnetic substrates using the MEC testing.

In this paper, a simplified iterative algorithm is proposed for the computation of inductance value under high working frequencies to cancel the lift-off effect. The simplified algorithm is based on the eddy-current thin-skin feature. That is, the inductance measured by the sensor is shown to be independent of the test piece (including the coating thickness) under high working frequencies. The lift-off is retrieved based on the proposed eddy-current thin-skin feature. Furthermore, it has been found that the inductance becomes insensitive to the lift-off at a certain value (termed as the lift-off insensitive inductance). The lift-off insensitive inductance is a quiescent value, which is shown to be material independent. Therefore, based on the retrieved lift-off and frequency of the lift-off insensitive inductance (LII) for different coatings, the thickness of coatings has been retrieved using an iterative method. Compared with our previous work on the thickness retrieval [47], an alternative sensor design with two sensing pairs is used, which has considered the sensitivities of sensing pairs with different lift-off on the retrieval of both lift-off and thickness retrieval (the lower sensing coil is sensitive to the coating thickness, while the upper one is sensitive to the lift-off spacing between the coil and test piece). Besides, a previous research work [47] focused on the thickness retrieval of single-layer nonferromagnetic materials. In this paper, the influence of ferromagnetic substrate is considered for the thickness retrieval of nonferromagnetic coatings. The ferromagnetic substrate is permeable (and even can be magnetized under large driving current or restrained eddy current under high-frequency skin effect) and thus affect the measured inductance and its sensitivities to different parameters [22] under different lift-offs and frequencies). Moreover, compared to [47], instead of retrieving the thickness under a random working frequency, a lift-off insensitive inductance feature is found in this paper (where the inductance is significantly less sensitive to the lift-off for a sensor-dependent inductance). The coating thickness is retrieved by referring to the corresponding frequency (merely determined by the test piece and significantly sensitive to the lift-off) of the lift-off insensitive inductance (merely determined by the sensor and independent of the test piece) on the multifrequency inductance spectrum. The measurement is based on the triple-stacked coil [22] sensor setup but has different dimensions and strategies of signal processing. The previous technique on the lift-off retrieval is based on the iterative method on conventional analytical model, whereas the proposed technique uses the thin-skin regime via simplified model (which only needs single frequency for the lift-off retrieval, and applies for the online measurement). Experiments on the inductance measurement of a ferrous dual-phase substrate with nonmagnetic coatings of different materials and thicknesses were carried out. The thickness of different coatings was retrieved based on the retrieved lift-off and frequency of LII (termed as the lift-off insensitive frequency) with an error of less than 3% for lift-offs up to 10 mm.

## 2. Analytical Algorithms

**Figure 1:**
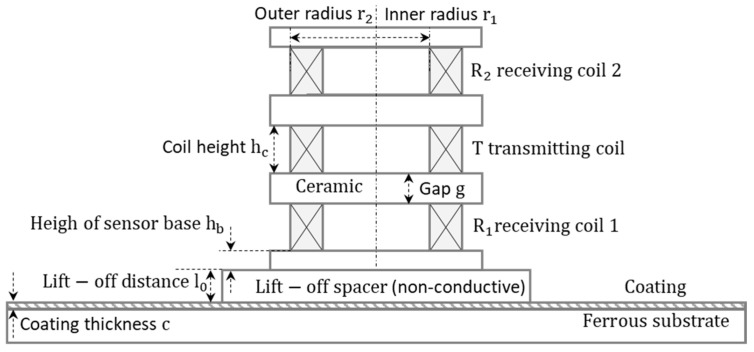
Circular coils above a dual-layer structure.

For eddy current sensing coils above the coated conductors (e.g., Figure 1), several parameters (including coating thickness c, lift-off spacing $l_{0}$ between sensor and test piece, electrical conductivities of coatings and substrate, and relative permeability of ferromagnetic substrate) affect the measured inductance ($L_{1}$ and $L_{2}$ from transmitting-receiving 1 ($T-R_{1}$) and transmitting-receiving 2 ($T-R_{2}$) sensing pairs). The aim is to find the function of retrieving the coating thickness c (i.e., $c=F\left(L_{1},L_{2}\right)$), where the function F needs to be calibrated.

To address the unwanted lift-off effect, the lift-off spacing is retrieved from the inductance $L_{2}$ ($T-R_{2}$ sensing pair) via a simplified function. Then, a lift-off insensitive inductance feature is proposed to retrieve the coating thickness c from the retrieved lift-off via $T-R_{1}$ sensing pair.

### 2.1. Original Formulas—Inductance of Coils above a Dual-Layer Conductive Structure

In Figure 1, the eddy current sensor consists of three identical circular coils. To fully receive the reflected magnetic flux from the specimen, two receiving coils are aligned co-axially with the transmitting coil.

Based on the Green’s functions, Dodd-Deeds formulas [48] have been massively applied for the analytical computation of mutual inductance between conductive samples and different sensor structures [40,41,42,49,50]. As shown in Figure 1, the inductance change (values due to the sample minus those for the sensor in the free space) for the transmitting-receiving 1 ($T-R_{1}$) and transmitting-receiving 2 ($T-R_{2}$) are given as following expressions.

(1) $$L_{1}\left(c,f\right)=K\int_{0}^{\infty }M_{1}\varphi d\alpha$$

(2) $$L_{2}\left(c,f\right)=K\int_{0}^{\infty }M_{2}\varphi d\alpha .$$

In (1) and (2), $L_{1}$ and $L_{2}$ vary with the frequency f of the exciting current and coating thickness c. α is the variable of integration, which is related to the wavenumber of the incident transverse electric (TE) planar electromagnetic wave [47,48,51,52]. φ is the material-dependent phase term for the mutual inductance. K is defined as follows.

(3) $$K=\frac{{\pi \mu }_{0}N^{2}\left(r_{2}+r_{1}\right)}{2h_{c}^{2}{\left(r_{2}-r_{1}\right)}^{2}}.$$

For the cross-sectional circular coil, $h_{c}$ is the coil height. N is the number of turns. $r_{1}$ and $r_{2}$ are the inner and outer radii of coil. $\mu_{0}$ denotes the vacuum magnetic permeability. $M_{1}$ and $M_{2}$ mainly control the magnitude of integrand for the mutual inductance in (1) and (2), respectively, which are merely determined by the dimension and structure of sensors.

(4) $$M_{1}=\frac{P^{2}\left(\alpha \right)}{\alpha^{6}}e^{-\alpha \left(h_{c}+g+2h_{b}+2l_{0}\right)}{\left(e^{-{\alpha h}_{c}}-1\right)}^{2}$$

(5) $$M_{2}=\frac{P^{2}\left(\alpha \right)}{\alpha^{6}}e^{-\alpha \left(3h_{c}+3g+2h_{b}+2l_{0}\right)}{\left(e^{-{\alpha h}_{c}}-1\right)}^{2}.$$

In (4) and (5),

(6) $$P\left(\alpha \right)=\int_{{\alpha r}_{1}}^{{\alpha r}_{2}}{\tau J}_{1}\left(\tau \right)d\tau .$$

$J_{1}$ is the first-order Bessel function of the first kind. τ is the variable of integration. $h_{b}$ is the height of the sensor base. $l_{0}$ is the lift-off distance between the sensor and test piece. g is the gap between coils.

By integrating the magnitude ($M_{1}$ or $M_{2}$) and phase term φ over the entire wavenumber domain, the whole contributions of inductance from TE planar electromagnetic waves can be derived.

As shown in Figure 1, for the dual-layer conductive structure, the phase of the integrand in (1) and (2) is expressed as.

(7) φ=Re((α+β1)(β1−β2)−(α−β1)(β1+β2)e2α1c(α−β1)(β1−β2)+(α+β1)(β1+β2)e2α1c).

In (7),

(8) $$\alpha_{1}=\sqrt{\alpha^{2}+j2{\pi \sigma }_{1}\mu_{1}\mu_{0}f}$$

(9) $$\beta_{1}=\frac{\sqrt{\alpha^{2}+j2{\pi \sigma }_{1}\mu_{1}\mu_{0}f}}{\mu_{1}}$$

(10) $$\beta_{2}=\frac{\sqrt{\alpha^{2}+j2{\pi \sigma }_{2}\mu_{2}\mu_{0}f}}{\mu_{2}}.$$

f is the working frequency of the current flowing in the transmitter coil. $\mu_{1}$ and $\mu_{2}$ are the relative permeability of top and bottom layers, respectively (i.e., the coating and substrate in Figure 1). $\sigma_{1}$ and $\sigma_{2}$ are the electrical conductivity of the coating and substrate. $\alpha_{1}$ and $\beta_{1}$ are related to the wavenumber of the TE planar electromagnetic wave within coatings. $\beta_{2}$ is related to the wavenumber of the TE planar electromagnetic wave within substrates [48,51], considering the effect of material inhomogeneities of different layers.

### 2.2. Proposed Method—Eddy-Current Thin-Skin Algorithms for the Retrieval of Lift-Off

For the case of the nonmagnetic coating on the ferromagnetic substrate, φ in (7) becomes,

(11) φ=Re((α+α1)(μ2α1−α2)−(α−α1)(μ2α1+α2)e2α1c(α−α1)(μ2α1−α2)+(α+α1)(μ2α1+α2)e2α1c).

In (11),

(12) $$\alpha_{2}=\sqrt{\alpha^{2}+j2{\pi \sigma }_{2}\mu_{2}\mu_{0}f}.$$

$\alpha_{2}$ is related to the wavenumber of the TE planar electromagnetic wave within substrates.

**Figure 2:**
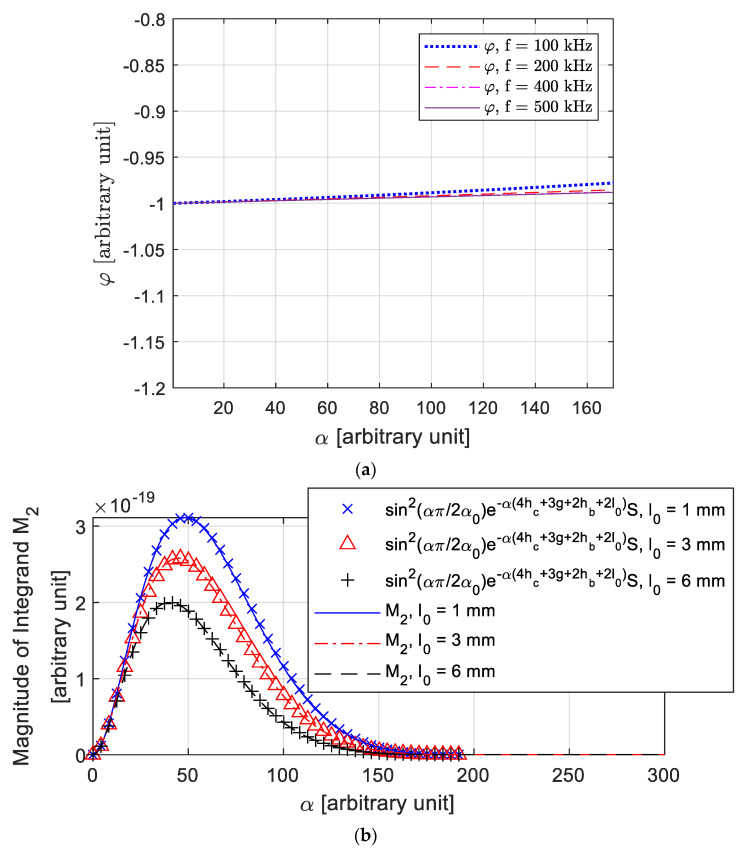

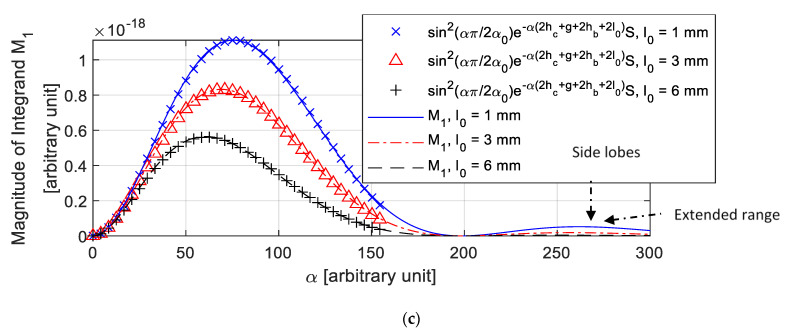
(**a**) Phase of the integrand in (1) and (2). (**b**) Matched function for the magnitude of the integrand in (2). (**c**) Side lobes occur in the magnitude of the integrand in (1); the range of α extends.

In Figure 2, it is found that under relatively high working frequencies (normally over 100 kHz for most of nonmagnetic metals), the phase φ changes very slowly compared to the magnitude part (In Figure 2b). Thus, φ can be approximated as a constant.

(13) φ=−1.

It is found that a larger lift-off of receiver coil could avoid side lobes in the magnitude of the integrand ($M_{1}$ in Figure 2c). Moreover, the effective range of α in $M_{2}$ is found to be narrower than in $M_{1}$, which results in a better high-frequency approximation in (13). Therefore, the lift-off is obtained from $T-R_{2}$ sensing pair.

Since the $J_{1}$ (the first-order Bessel function of the first kind) is similar to the sinusoidal function with a decay factor. In Figure 2b, for the magnitude part of integrand in (2), it is found that the Bessel series $\frac{P^{2}\left(\alpha \right)}{\alpha^{6}}e^{{\alpha h}_{c}}{\left(e^{-{\alpha h}_{c}}-1\right)}^{2}$ can be well fitted by a sinusoidal function $\sin^{2}\left(\frac{\alpha \pi }{2\alpha_{0}}\right)$. $\alpha_{0}$ is a sensor-dependent factor, which is determined by parameters $h_{c}$, $r_{1}$, and $r_{2}$. Hence, $M_{2}$ can be expressed as,

(14) $$M_{2}={\mathrm{Se}}^{-\alpha \left(4h_{c}+3g+2h_{b}+2l_{0}\right)}\sin^{2}\left(\frac{\alpha \pi }{2\alpha_{0}}\right).$$

In (14), S is a normalisation factor between the Bessel function (for variable α) and the sinusoidal function (for variable α). S is derived from the ratio between two functions at the peak of sinusoidal function when α arrives at $\alpha =\alpha_{0}.$

(15) $$S=\frac{P^{2}\left(\alpha_{0}\right)}{\alpha_{0}^{6}}e^{\alpha_{0}h_{c}}{\left(e^{-\alpha_{0}h_{c}}-1\right)}^{2}.$$

It can be seen in (15) that S is determined by the sensor-dependent constant $\alpha_{0}$ instead of the wavenumber valuable α.

Substituting (14) into (2), the high-frequency inductance becomes,

(16) $$L_{2}\left(c,f\right)=-K\int_{0}^{2\alpha_{0}}{\mathrm{Se}}^{-\alpha \left(4h_{c}+3g+2h_{b}+2l_{0}\right)}\sin^{2}\left(\frac{\alpha \pi }{2\alpha_{0}}\right)d\alpha .$$

Assume $\left(x=4h_{c}+3g+2h_{b}+2l_{0}\right)$, evaluating the integral yields,

(17) $$L_{2}\left(c,f\right)=-\frac{\pi^{2}\mathrm{KS}\left(1-e^{-2\alpha_{0}x}\right)}{2x\left(\alpha_{0}^{2}x^{2}+\pi^{2}\right)}.$$

In (17), $e^{-2\alpha_{0}x}\ll 1$ as $2\alpha_{0}x\gg 1$. Thus,

(18) $$2x\left(\alpha_{0}^{2}x^{2}+\pi^{2}\right)L_{2}\left(c,f\right)+\pi^{2}\mathrm{KS}=0.$$

Assume the solution of x in the function (18) is $x_{0}$, the lift-off is,

(19) $$l_{0}=\frac{x_{0}-3g}{2}-2h_{c}-h_{b}.$$

### 2.3. Proposed Method—Iterative Algorithms Based on a Lift-Off Insensitive Inductance for the Retrieval of Coating Thickness

**Figure 3:**
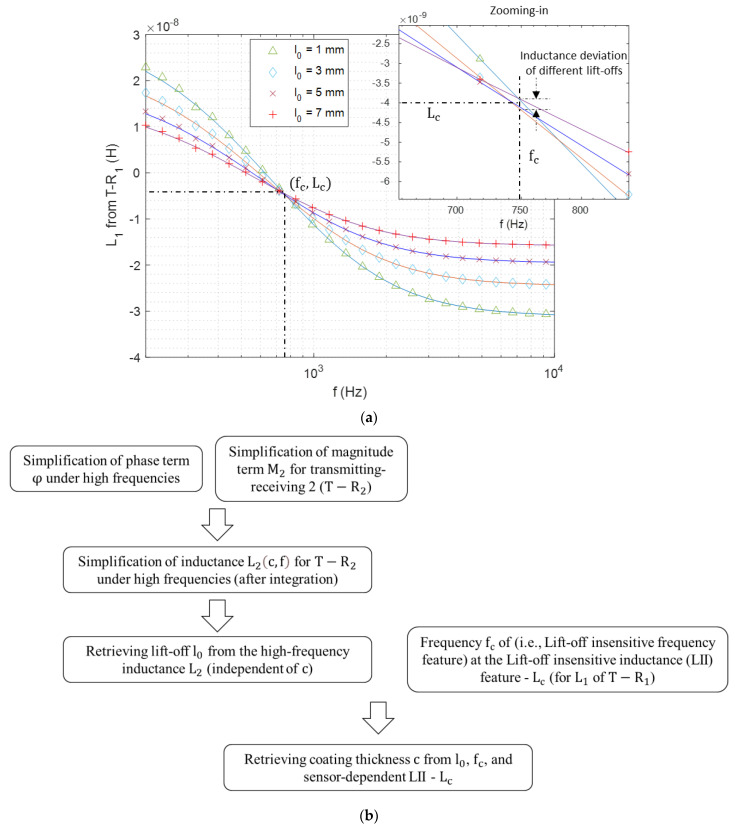
(**a**) Swept-frequency inductance curves of different lift-off distances (solid lines—analytical results via Equation (1), markers—experimental results) for $T-R_{1}$ above the substrate (DP 1000) with aluminium coating of 0.3 mm (Table 1). (**b**) Algorithmic flowchart.

As receiver 1 ($R_{1}$) is closer and more sensitive to the coating, the signal of $T-R_{1}$ sensing pair is used for the retrieval of coating thickness. As can be observed from Figure 3a, swept-frequency inductance curves with different lift-offs nearly intersect at an inflection point. It is found that the inductance of the intersected point is independent of the test piece (including the thickness of coatings). The inductance and frequency of the intersected point are termed as the lift-off insensitive inductance (LII) and lift-off insensitive frequency (LIF), respectively. Thus, the thickness of coatings can be retrieved by referring to the LIF feature. In practical measurement, inductance curves may intersect at multiple cluster points. Consequently, LII is the least-squares value of the inductance for different lift-offs under LIF. Moreover, LIF is selected when the inductance deviation of different lift-offs under a single frequency reaches its lowest value.

Referring to the signal processing method based on the modified Newton-Raphson method [22], the thickness of coatings can be restored in an iterative manner.

(20) $$c=\Delta c+c_{r}.$$

$c_{r}$ is the reference coating thickness. The increment term Δc is defined as,

(21) $$\Delta c=J^{-1}\left(L_{1}\left(c_{r},f_{c},l_{0}\right)-L_{c}\right).$$

$L_{1}\left(c_{r},f_{c},l_{0}\right)$ denotes the inductance expressed in Equation (1) for the reference coating thickness ($c_{r}$), LIF ($f_{c}$), and derived lift-off $l_{0}$. $L_{c}$ is the sensor-dependent LII. J is the Jacobian matrix, which denotes the inductance sensitivity with respect to $c_{r}$.

(22) $$J=\frac{L_{1}\left(c_{r},f_{c},l_{0}\right)-L_{1}\left(c_{r}+{\rho c}_{r},f_{c},l_{0}\right)}{\rho c}.$$

In (22), ρ is a residual value (ρ is assigned as 0.01 here).

Figure 3b depicts the algorithmic flow of strategies on retrieving the coating thickness from the measured inductance of eddy current sensing coils.

## 3. Experiments

To investigate the inverse algorithm from (18) to (22), experiments have been conducted on the inductance measurement of the triple-coil sensor above the ferrite-austenite dual-phase (DP) 1000 substrate with coatings of different nonmagnetic materials and thicknesses (Table 1). Different thicknesses of coatings are achieved by stacking a series of thin foils. Since the eddy current is parallel to the coating, the induced eddy current is mainly parallel to the coating layers. Consequently, impedance interferences between foils are neglectable.

**Figure 4:**
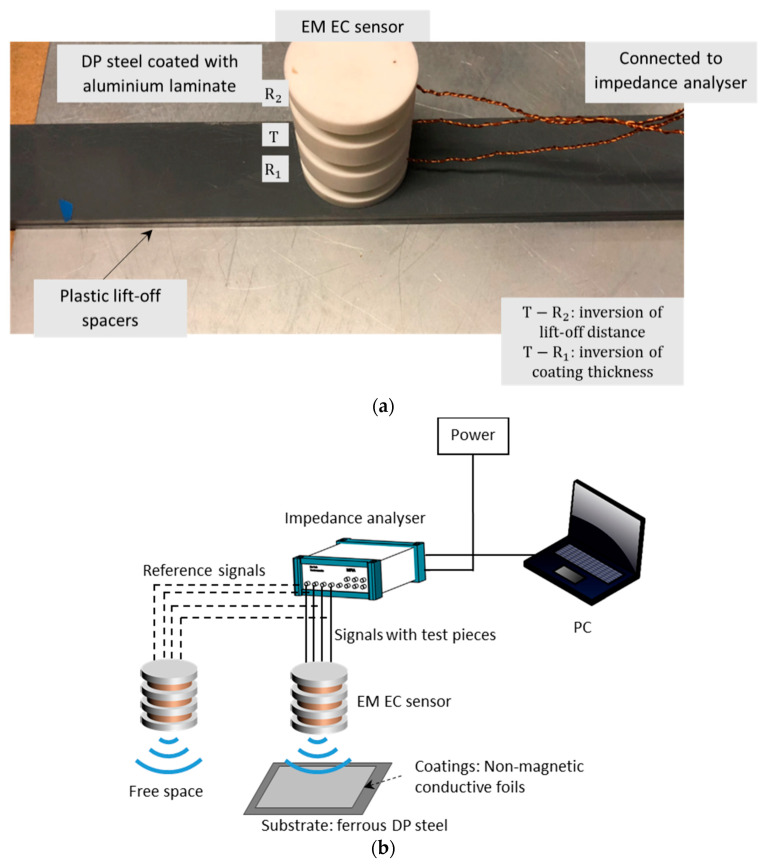
Experimental setup (**a**) sensor placed on the test piece (**b**) sensor connected to the measurement system (impedance analyser).

As shown in Table 2 and Figure 4, the frame of eddy-current sensor is designed as a ceramic structure, which contains three coaxial circular buckets. Three identical coil windings are wound seamlessly in the ceramic slot. In the measurement, the eddy-current sensor is placed on layers of plastic spacers to mock the lift-off effect.

In Figure 4, the sensor is connected to the impedance analyser for the measurement of swept-frequency inductance for both free space and above the test piece. The inductance change is the value due to the sample minus that for the sensor in the free space. Considering the SNR and ambient effects (including the resonant/proximity/parasitic effect, and Barkhausen noise effect—where the permeability of ferrous substrate becomes frequency dependent) under low and high working frequencies, respectively, the frequency range is set from 200 Hz to 500 kHz.

## 4. Result and Analysis

### 4.1. Retrieval of Lift-Off Distance

**Figure 5:**
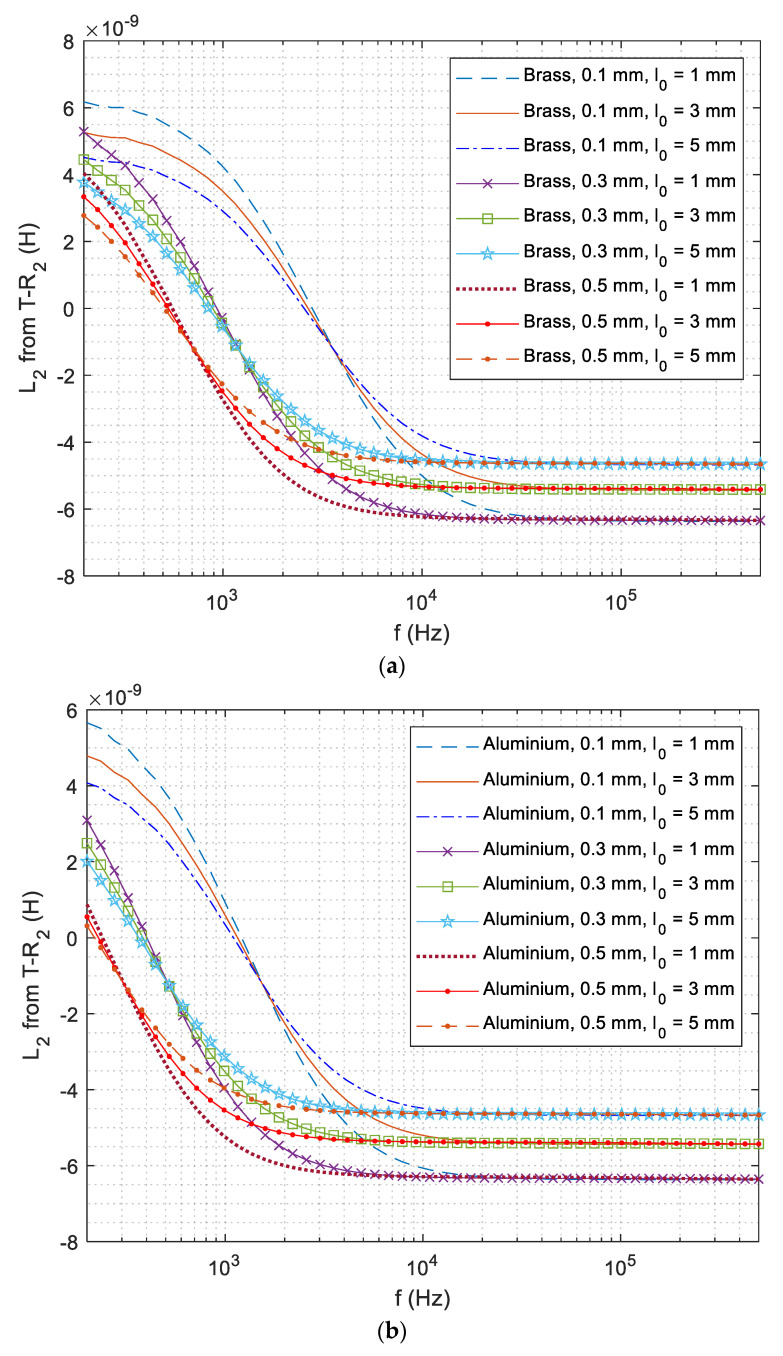
Experimental swept-frequency inductance curves of different lift-off distances for $T-R_{2}$ above the conductive coating (on DP 1000 steel) with different thicknesses: (**a**) brass and (**b**) aluminium.

Figure 5 shows the experimental swept-frequency inductance curve from $T-R_{2}$ sensing pair. Due to the magnetic permeability of ferrous substrate, the inductance curve starts from a positive value instead of zero. With increasing frequency, the inductance crosses zero (zero-crossing frequency feature reported in [49], instead of the point of intersection) and gradually becomes stable especially over 40 kHz, where inductance curves of different coating thicknesses converge. As the overall conductivity of aluminium coatings with DP 1000 substrate is higher than that of brass coatings with DP 1000 substrate, the zero-crossing frequency and whole inductance curve shift left [49]. Moreover, the inductance curve is mainly determined by the lift-off distance between the sensor and test piece for working frequencies over 40 kHz. Considering the effect of other metals, the lift-off distance is retrieved from the inductance under working frequencies over 100 kHz.

**Figure 6:**
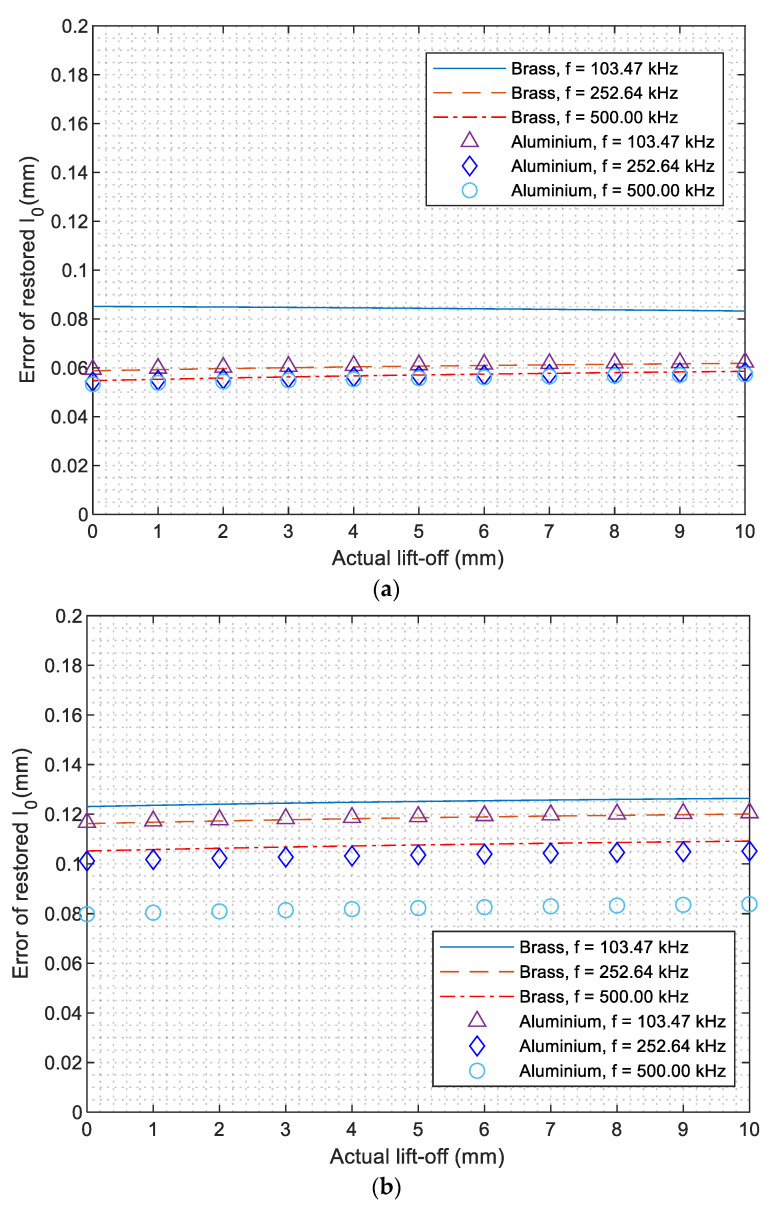

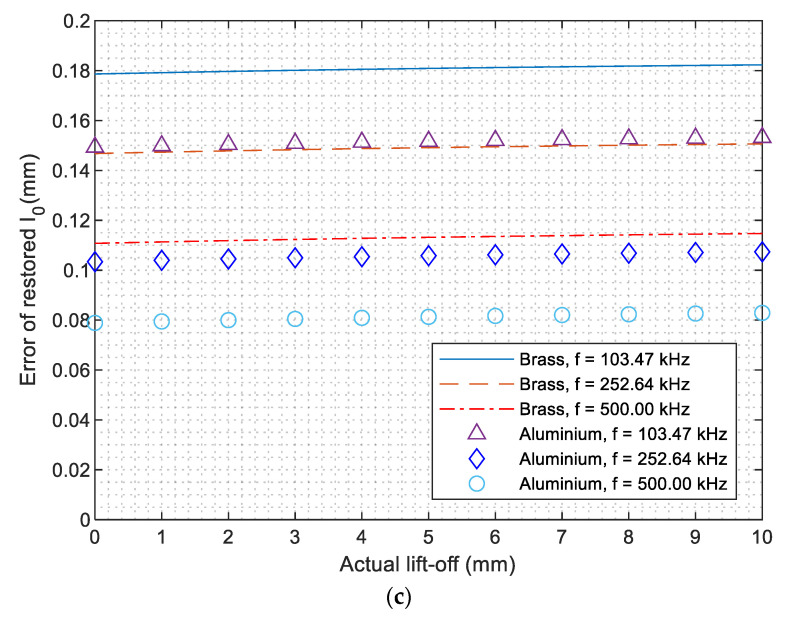
Error of the retrieved lift-off distance for coatings with thickness of (**a**) 0.1 mm, (**b**) 0.3 mm, and (**c**) 0.5 mm.

Figure 6 shows the error of retrieved lift-off from the measured high-frequency inductance using proposed algorithms (18) and (19). It can be observed that the error of inversed lift-off slightly increases with the actual spacing distance between the sensor and test piece. Overall, the lift-off is slightly overestimated for all the coatings, which is caused by the small deviation of the phase term φ approximation in Figure 2a and Equation (13) and omitting of exponential term $e^{-2\alpha_{0}x}$ from (17) to (18). As the change rate of φ for thicker coatings is slightly higher than that of thin coatings, the phase term φ in Equation (13) is more underestimated. Consequently, the error of inverse lift-off generally increases with coating thickness. Since the approximation of phase term φ in Equation (13) achieves a better performance under higher working frequencies (as Figure 2a depicts), the inversed lift-off is more accurate from the inductance under 500 kHz. Therefore, the inverse of coating thickness in the following section is based on the inversed lift-off under 500 kHz. For coatings of different materials and thickness, the error of inversed lift-off has been controlled within 0.2 mm for different coatings thicknesses.

### 4.2. Effect of High Frequency on Lift-Off Retrieval

**Figure 7:**
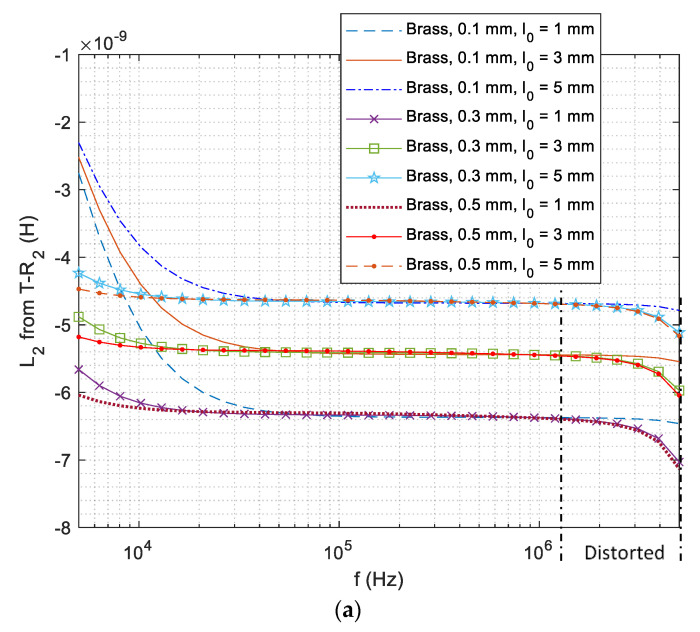

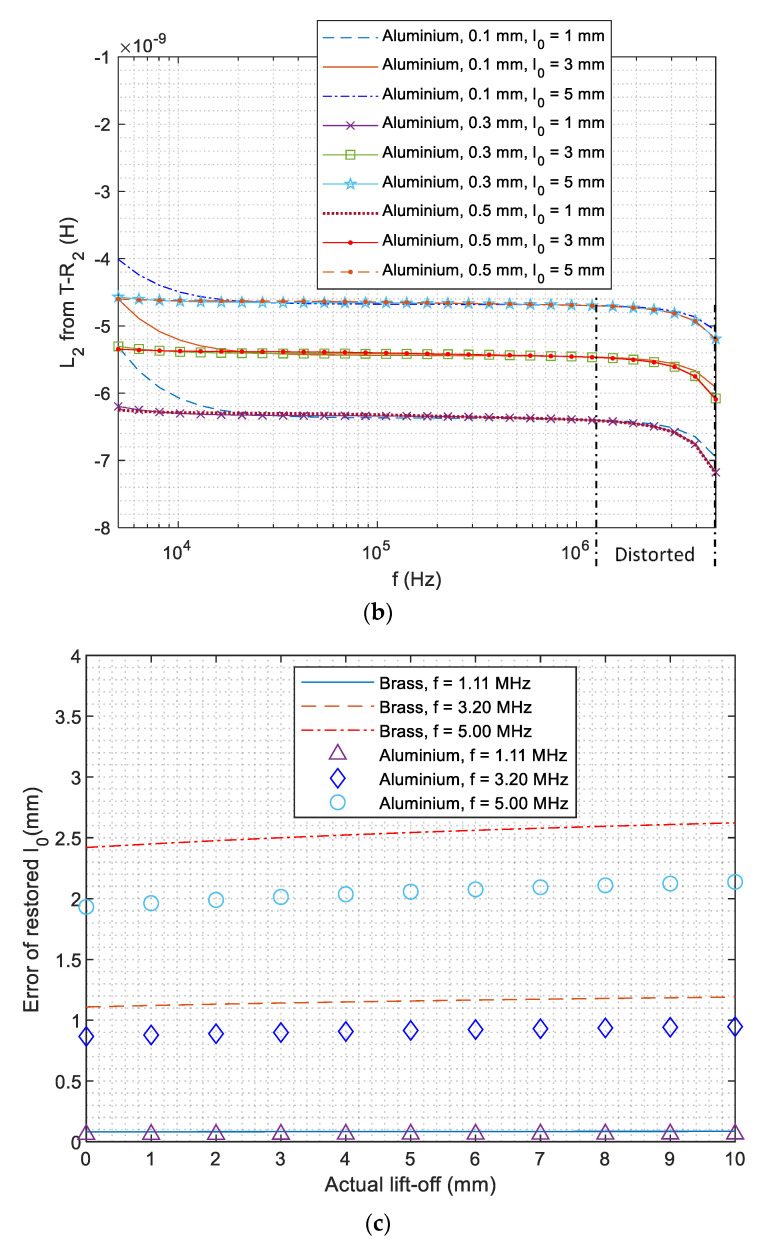
Distorted swept-frequency inductance (for high working frequencies up to 5 MHz) of different lift-off distances for $T-R_{2}$ above the conductive coating (on DP 1000 steel) with different thicknesses: (**a**) brass, (**b**) aluminium, and (**c**) error of the retrieved lift-off distance for coatings with thickness of 0.3 mm.

Figure 7a,b shows the swept-frequency inductance of sensing pair for high working frequencies up to 5 MHz. It can be observed that the inductance curve gradually diverges from the constant value and becomes distorted. Such measurement is caused by various factors, including the resonant/proximity/parasitic effect of coil windings under high frequency (fringe effect of excitation current), and Barkhausen noise effect (where the ferromagnetic domains of the substrate surface are magnetized by the restrained eddy current under the high-frequency skin-effect). Figure 7c illustrates the error of the retrieved lift-off for coatings with a thickness of 0.3 mm. A higher working frequency (e.g., 5.0 MHz) results in a more distorted inductance and larger error for the lift-off retrieval.

### 4.3. Retrieval of Coating Thickness

**Figure 8:**
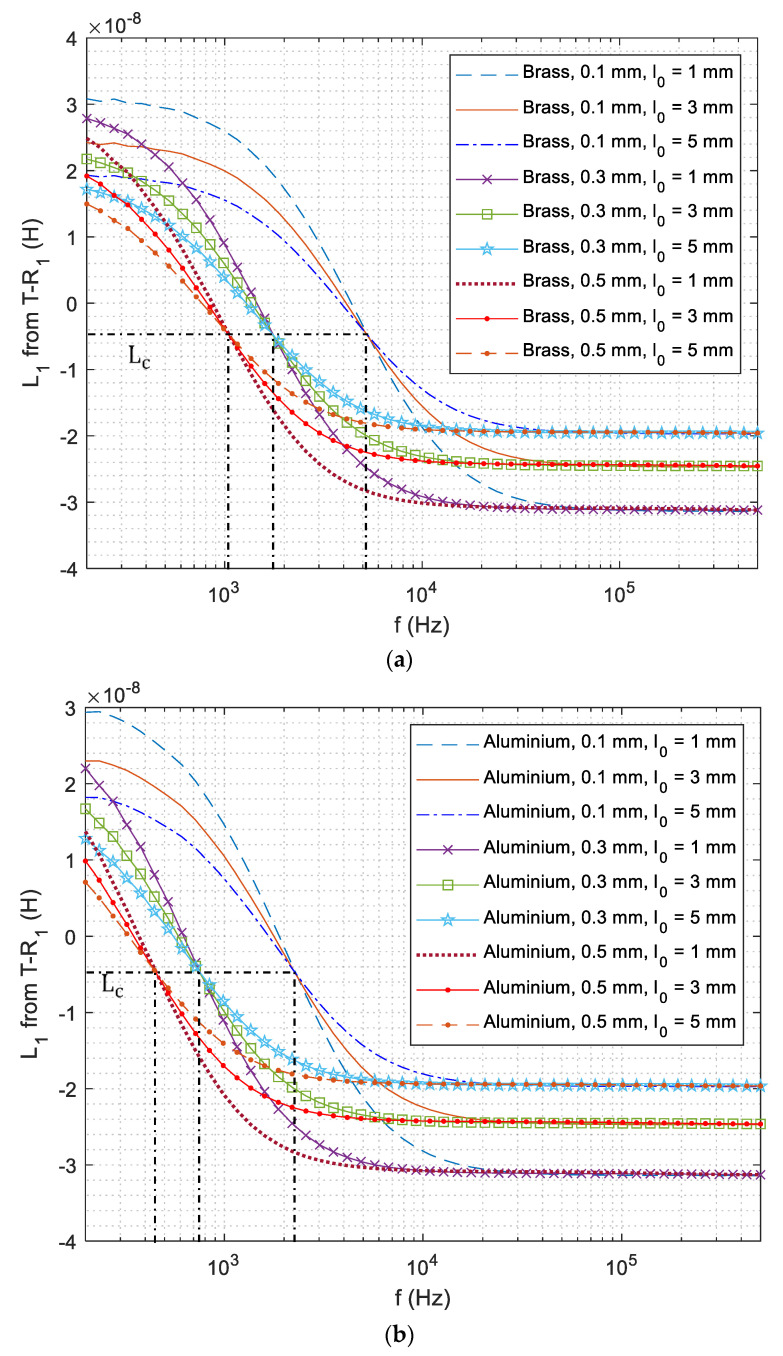
Experimental swept-frequency inductance curves of different lift-off distances for $T-R_{1}$ above the conductive coating (on DP 1000 steel) with different thicknesses: (**a**) brass and (**b**) aluminium.

Figure 8 illustrates the measurement of swept-frequency inductance curve from $T-R_{1}$ sensing pair, which follows a similar trend in Figure 5. It can be observed that inductance curves of different lift-offs converge at the point (clusters). Moreover, curves of different coating thicknesses and materials share the same LII. Compared to the swept-frequency inductance curve from $T-R_{2}$ in Figure 5, the LIF of the lift-off insensitive point and inductance curve slightly shifts towards high frequencies. Since the inductance curve slightly fluctuates under lower frequencies due to the poor SNR, the inverse of coating thickness from the LIF feature achieves a better performance from $T-R_{1}$ sensing pair (compared to $T-R_{2}$ sensing pair). Compared to the previous zero-crossing frequency feature [49] where the inductance crosses zero, the LIF is less sensitive to the lift-off variations.

**Figure 9:**
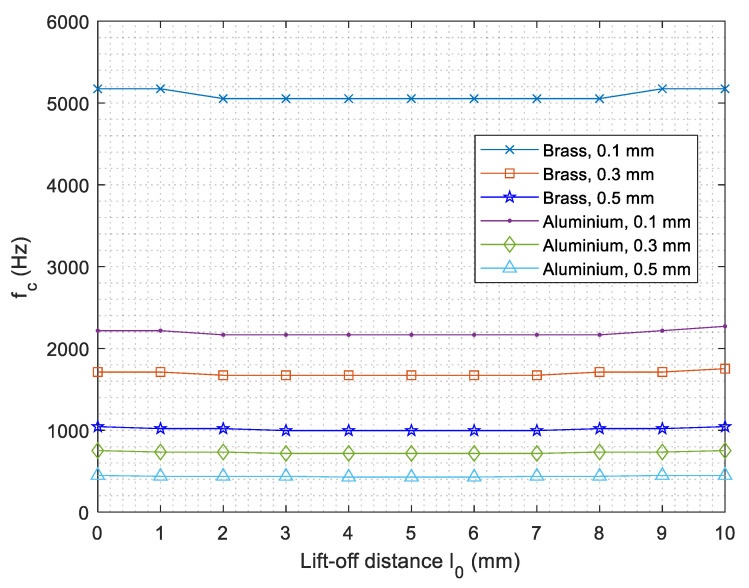
Lift-off insensitive frequency versus lift-off distance for different coatings.

Figure 9 exhibits corresponding frequencies (LIF) of the LII for different coatings under different lift-offs. It can be observed that LIF slightly fluctuates with increased lift-offs. Moreover, either a highly conductive or thinner coating will render an increased LIF.

Furthermore, parameters including the LIF and retrieved lift-off are served as the input for the reconstruction of coating thickness using iterative algorithms from (20) to (22).

**Figure 10:**
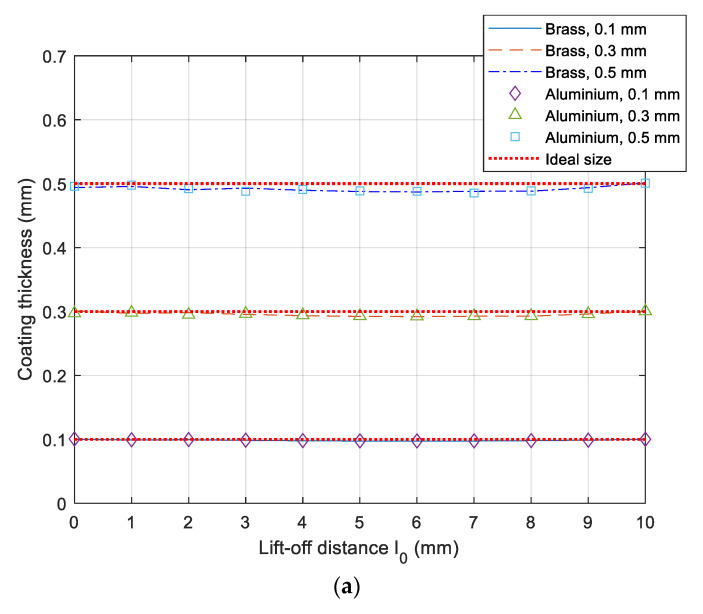

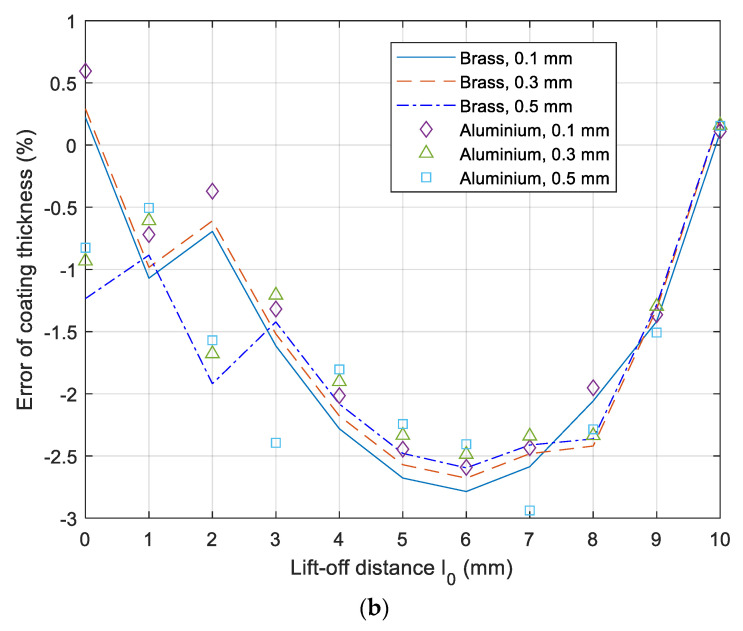
Inverse of coating thickness versus lift-off distance: (**a**) absolute value and (**b**) error.

In Figure 10, owing to different inductance sensitivities with respect to different thicknesses, electrical conductivities, and lift-offs [22], the retrieved coating thickness is sensitive to the lift-off variation (compared to inversed lift-off in Figure 6 and LIF in Figure 9). As the lift-off increases, the calculated coating thickness drifts away and then converges to its actual size. Overall, the inverse error for the thickness of different coatings on the DP 1000 steel is controlled within 3% for lift-off up to 10 mm.

## 5. Conclusions

A simplified iterative algorithm is proposed for the computation of the inductance of circular coils above ferrous substrate with nonmagnetic coatings under high working frequencies. In this regime, either the phase term or inductance is insensitive to the property of the dual-layer conductors (including the thickness of coatings). The lift-off is retrieved from the high-frequency inductance based on the proposed algorithm. Based on the LII and LIF features, where swept-frequency inductance curves of different lift-off intersect at one point or converge at point clusters, the thickness of different coatings is calculated in an iterative manner. Considering the sensitivities of inductance with respect to lift-offs and sample parameters, the measurement is based on two different coil-sensing pairs. The sensor consists of two receiving coils ($R_{1}$ and $R_{2}$) of different lift-offs and one transmitting coil (T) in the middle, with the top sensing pair $T-R_{2}$ more accurate on the lift-off retrieval and the bottom one $T-R_{1}$ more sensitive to the sample parameters. Experiments show that the calculation is independent of the lift-off distance variations, with a maximum deviation of 0.18 in 10 mm range. Moreover, with the referred LIF and compensated lift-off, the retrieved coating thickness can be controlled within a deviation of 3%. For different nonmagnetic coatings and ferrous substrates, the LII is a constant factor determined by the dimension and geometry of the sensor. The property of nonmagnetic coatings (e.g., thickness) and ferrous substrate (e.g., magnetic permeability) effects can thus be retrieved by referring to the corresponding LIF. The proposed method is based on the simplification of inductance algorithms using eddy-current thin-skin effect, which significantly relieved the cumbersome calculations of integrations for the retrieval. Thus, based on the eddy-current thin-skin effect under high frequencies, the proposed formula is used as the embedded algorithm for the online retrieval of lift-offs. Compared to previous techniques of compensating the lift-off error, the lift-off retrieval is directly retrieved from the impedance and used for the retrieval of coating thickness. Moreover, the lift-off range that can be retrieved is extended from 3 to 10 mm, while the error of thickness retrieval is still within a deviation of 3%.

## Figures and Tables

**Table 1:** Properties of samples.

	Electrical Conductivity (MS/m)	Relative Magnetic Permeability	Thickness (mm)
Substrate—DP 1000	3.81	122	4.0
Coating—brass	15.9	1	0.1, 0.3, 0.5
Coating—aluminium	36.9	1	0.1, 0.3, 0.5

**Table 2:** Properties of sensor structures and excitation signals.

Parameters	Value
Inner radius $r_{1}$ (mm)	19.0
Outer radius $r_{2}$ (mm)	19.6
Turns N	20
Gap g (mm)	10.0
Coil height $h_{c}$ (mm)	6.0
Heigh of sensor base $h_{b}$ (mm)	4.0
Lift-offs $l_{0}$ (mm)	1.0:1.0:10.0
Working frequency	200 Hz~500 kHz
Lift-off insensitive inductance $L_{c}$ (H)	$-4\times 10^{-9}$

## Data Availability

Data of this research is available upon request via corresponding author.
